# The lung microbiome in HIV-positive patients with active pulmonary tuberculosis

**DOI:** 10.1038/s41598-022-12970-3

**Published:** 2022-05-28

**Authors:** Veronica Ueckermann, Pedro Lebre, Janri Geldenhuys, Ebrahim Hoosien, Don Cowan, Luricke Janse van Rensburg, Marthie Ehlers

**Affiliations:** 1 Department of Internal Medicine, University of Pretoria, Pretoria, South Africa; 2 Centre of Microbial Ecology and Genomics, Department of Biochemistry, Genetics and Microbiology, University of Pretoria, Pretoria, South Africa; 3 Department UP-Ampath Translational Genomics Initiative, University of Pretoria, Pretoria, South Africa; 4 Department Medical Microbiology, Ampath Laboratories and University of Pretoria, Pretoria, South Africa; 5 Department Medical Microbiology, University of Pretoria, Pretoria, South Africa

**Keywords:** Microbiology, Diseases

## Abstract

Tuberculosis poses one of the greatest infectious disease threats of our time, especially when associated with human immunodeficiency virus (HIV) infection. Very little data is available on the lung microbiome in pulmonary tuberculosis (PTB) in HIV-positive patients. Three patient cohorts were studied: (i) HIV-positive with no respiratory disease (control cohort), (ii) HIV-positive with pneumonia and (iii) HIV-positive with PTB. Sputum specimens were collected in all patients and where possible a paired BALF was collected. DNA extraction was performed using the QIAamp DNA mini kit (QIAGEN, Germany) and extracted DNA specimens were sent to Inqaba Biotechnical Industries (Pty) Ltd for 16S rRNA gene sequence analysis using the Illumina platform (Illumina Inc, USA). Data analysis was performed using QIMME II and R Studio version 3.6.2 (2020). The lung microbiomes of patients with PTB, in the context of HIV co-infection, were dominated by *Proteobacteria*, *Firmicutes*, *Actinobacteria* and *Bacteroidetes*. Loss of biodiversity and dysbiosis was found in these patients when compared to the HIV-positive control cohort. Microbial community structure was also distinct from the control cohort, with the dominance of genera such as *Achromobacter, Mycobacterium, Acinetobacter, Stenotrophomonas* and *Pseudomonas* in those patients with PTB. This is the first study to describe the lung microbiome in patients with HIV and PTB co-infection and to compare findings with an HIV-positive control cohort. The lung microbiomes of patients with HIV and PTB were distinct from the HIV-positive control cohort without PTB, with an associated loss of microbial diversity.

## Introduction

Tuberculosis (TB) poses a major health threat worldwide, causing an estimated two million deaths annually according to the WHO (2020)^1^. The morbidity and mortality associated with TB is particularly severe in sub-Saharan Africa, where it is frequently associated with concomitant Human Immunodeficiency Virus (HIV) infection^1,2^.

The role of microbiota in health and disease has been well-characterised in various body sites^3–7^, using high-throughput sequencing technologies^8^. Although initially considered as a ‘sterile’ site, the healthy lung has been found to contain members of the phyla *Bacteroides, Firmicutes* and *Proteobacteria*^9,10^.

The lung microbiome is altered by acute and chronic disease, which alters the local conditions and inflammatory milieu of the lung^11^. Such alteration has been observed in chronic obstructive airways disease (COPD)^12^, asthma^13^, cystic fibrosis (CF)^14^, pneumonia^15^ and HIV infection^16^. When considering the lung microbiome in PTB in populations with a high prevalence of HIV, the effect of HIV itself on the microbiome should be considered.

The lung microbiome in TB has been less well characterized, with a few published studies^17–23^, none in the context of HIV. Loss of microbial diversity and the onset of dysbiosis has been illustrated in the lung microbiomes of patients with PTB, when compared with healthy controls^22^.

This study was the first to describe the lung microbiome of sputum and BALF specimens using 16S rRNA gene sequencing of three patient cohorts consisting of: (i) patients with HIV but no pulmonary disease (control cohort), (ii) patients with HIV and pneumonia, iii) patients with HIV and TB.

## Methods

### Sample collection

This study was conducted on adult (18 years and older) HIV-infected patients, presenting to Tertiary Academic Hospitals in Pretoria, South Africa, with the diagnosis of pneumonia. Participants for the control cohort were recruited from the Antiretroviral clinic, where patients were referred for initiation of antiretroviral therapy. Patients who had antibiotic exposure in the preceding 6 weeks were excluded from the study. Written informed consent was obtained from all participants. Ethical approval to perform the study was obtained from the Research Ethics Committee, Faculty of Health Sciences, University of Pretoria (REC number 265/2017). All components of the study were performed in alignment with local and international guidelines and regulations, and the research adhered to the principles set out in the Declaration of Helsinki.

Sputum was obtained from all patients enrolled in the study (n = 71), using a lung flute (Medical Acoustics, USA)^23^. When possible bronchoscopy was performed to collect an additional BALF specimen.

### Sample processing and DNA sequencing

Respiratory specimens in those with pneumonia were sent for diagnostic testings: microscopy, culture and antibiotic susceptibility testing, Gene Xpert (Cepheid, South Africa), *Pneumocystis* PCR (Fast Track diagnostics, Siemens, Germany) at the National Health Laboratory Service (NHLS). The diagnosis of HIV was confirmed in all the participants, by a positive fourth generation ELISA test (Siemens healthcare diagnostics, Germany).

The DNA extraction was done with the QIAamp DNA mini kit (QIAGEN, Germany) according to the manufacturer manual for bacteria and extracted DNA samples were sent to Inqaba biotechnical industries (pty) ltd, South Africa, for sequencing. A sequencing library was prepared by random fragmentation of the DNA sample, which was followed by 5’and 3’adapter ligation. The adapter-ligated fragments were PCR amplified using universal primer pairs (341F and 785R, targeting the V3 and V4 regions of the 16S rRNA gene).

**Figure 1:**
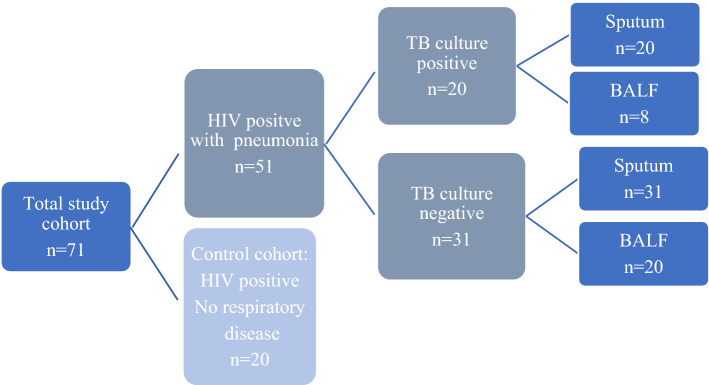
Participant cohorts included in the study.

Based on the culture results patients were separated into three cohorts: (i) HIV-positive control cohort with no respiratory disease (“HIV” cohort), (ii) HIV-positive with pneumonia but TB cultures negative (“Pneumonia” cohort) and (iii) HIV-positive with pneumonia and TB cultures positive (“TB”cohort). These cohorts are schematically represented in Fig. 1.

Three specimens that consisted only of 0.9% saline were included as negative controls for quality assurance purposes. These specimens underwent all the processing procedures, including DNA extraction and sequencing.

### Statistical analysis

Demographic and laboratory were summarized as a meta-data table. The descriptive data averages were calculated as percentages and mean values between the three cohorts were compared by an Analysis of Variance (ANOVA). Percentages were compared by the Fisher Exact test.

Sequencing data was analysed using the QIIME 2^25^ pipeline. DADA2^26^ was used for quality control and to infer unique sample sequences (amplicon sequence variants, ASVs) from the sequencing data. A quality score threshold of 15 was used to filter out low quality and chimeric reads, with positions 280 and 210 chosen for 3’-terminus trimming of forward and reverse reads, respectively. Clustered ASVs were subsequently assigned to taxonomic groups using the SILVAv138 prokaryotic database^27^.

Statistical analysis of assigned reads was performed in R Studio version 3.6.2 (2020)^28^. Before statistical analysis, the ASV table was filtered to remove ASVs that occurred in less than 90% of specimens. In addition, reads present in the negative controls, which were likely to occur due to contamination from the sampling protocol, DNA extraction or sequencing were used to filter out ASVs that are not biologically related to the patients. Specimens were grouped by cohort, using the patient data previously recorded.

Relative abundance values were calculated as fractions of the total sample count for the phyla representing 99% of the reads. The Deseq2^29^ pipeline was used to compare sample communities and calculate taxa that were over-represented in some cohorts when compared to the others.

Alpha diversity was calculated using the Observed, Inverse Simpson (richness) and Shannon (evenness) indices. The Shapiro test was performed to test the normality of distribution of the resulting indexes. Normally distributed data was compared using the ANOVA test^30^, while non-normal data was compared with the Kruskal–Wallis test^31^. Pair-wise comparisons were made using the Tukey^32^ test for normal data and the Wilcox^33^ test for non-normal data. A p-value of < 0.05 was considered significant.

Beta diversity was calculated using DeSeq2^29^ by the Jaccard^34^ and Bray Curtis^35^ indices and plotted in the component analysis (PCoA) -graph.

### Ethical approval and consent to participate

Before this study was commenced ethical approval of the research protocol was obtained from the University of Pretoria, Faculty of Health Sciences Research Ethics Committee (ref 265/2017). All patients enrolled in the study first underwent a process of voluntary signed informed consent.

## Results

### The patient population studied

During the period of February 2018 to January 2019, a total of 71 HIV-positive patients were enrolled in the study: 20 HIV, 31 Pneumonia (TB negative) and 20 with TB. Twenty-eight (20 in the Pneumonia cohort and eight in TB cohort) underwent bronchoscopy for the collection of BALF.

The details of the three cohorts of patients enrolled in the study are presented in Table 1. The patients had a mean age of 39 years and all were of African ethnicity. Fifty-one percent of participants were female and 49% were male, with no significant differences between the three cohorts.

**Table 1:** Baseline characteristics of participants enrolled in the study.

Variable	HIV-positive controls (n = 20)	Pneumonia TB negative (n = 31)	Pneumonia TB positive (n = 20)	p value
**Age (years)**				
Mean (± SD)	37.5 (± 12.4)	39.7 (± 13.8)	40.0 (± 13.8)	0.787
Min/Max	22/78	19/65	20/63	
**Gender**				
Male, n( %)	11 (55.0)	16 (51.6)	8 (40)	0.535
Female, n(%)	9 (45.0)	15 (48.4)	12 (60)	
Previous TB n(%)	1 (5.0)	4 (12.9)	5 (25)	0.153
Smoker n(%)	6 (30.0)	4 (12.9)	4 (20)	0.335
Biomass fuel exposure n (%)	4 (20.0)	4 (12.9)	3 (15)	0.774
**Inhaled medication**				
Corticosteroids n (%)	0 (0)	0 (0)	0 (0)	0.762
Beta-agonist n (%)	1 (5)	1 (3.2)	0 (0)	
**HIV Viral load (copies/mL)**				
n	6	16	10	0.188
Mean	27 561	242 190	420 036	
Min/Max	730/131 556	undetectable/1 120 775	34,912/1 578 513	
**CD4 (cells/mm**^**3**^**)**				
Mean	262 (± 195)	183 (± 142)	127 (± 130)	0.025*
Min/Max	84/942	3/486	1/363	
**TB culture**				
Positive	–	–	20 (100)	< 0.001*
Negative	20(100)	31(100)	–	
**PJP PCR**				
Positive (%)	–	7(22.6)	3(15)	0.319
Negative (%)	–	25(77.4)	17(85)	
**ARVs**				
Yes (%)	–	12 (38.7)	6 (30)	0.002*
No (%)	20 (100)	19 (61.3)	14 (70)	
**Severity of illness**				
CURB-65 (avg)	–	2,38	2.43	0.671
ACHU (avg)	–	2.56	2.34	0.459

*Statistically significant.

Previous tuberculosis was noted in 15% (10/71) participants, one (5%) in the HIV cohort, 4 (12.9%) in the pneumonia cohort and 5 (25%) in the TB cohort (not statistically significant). There were no statistically significant differences between the HIV, pneumonia and TB cohorts in respect of previous TB infection (p = 0.153), smoking status (p = 0.335) or biomass fuel exposure (p = 0.774).

There was a statistically significant difference between the mean CD4 counts between the three cohorts (p = 0.025). Pair-wise comparisons showed that the mean CD4 counts for the HIV cohort (262 cells/mm^3^), pneumonia cohort (183 cells/mm^3^) and TB cohort (127 cells/mm^3^) differed significantly (t test, p = 0.007). The HIV viral load was only available for 45% (32/71) of the participants. Despite the mean viral load being higher in the TB cohort, no statistically significant difference in HIV viral load between the cohorts was detected (p = 0.188).

A statistically significant difference between the use of antiretrovirals (ARV’s) was seen in the three cohorts (p = 0.002). None of the participants in the HIV cohort were on antiretroviral therapy. The ARV use between the TB positive and TB negative pneumonia cohorts did not differ significantly (p = 0.557). Notably, only 26.7% (19/71) of the study cohort in total were on ARV’s. The difference in severity of pneumonia, as measured by both the CURB-65 and the ACHU scores, was not statistically significant between the pneumonia and TB cohorts. The baseline characteristics of the study participants are detailed in Table 1.

### The lung microbiome defined by 16S rRNA sequencing

Due to the nature of the amplicon sequencing methodology used in this study, ASVs could be confidently assigned to genus as the lowest taxonomic rank. Quality and chimeric filtering resulting in a total of 2 089 648 reads were analysed. Thirty-four phyla and 396 genera were identified across all specimens. The dominant microbiota were defined as phyla with a mean relative abundance > 1% across all samples. Five dominant phyla across all specimens were: *Proteobacteria, Firmicutes, Actinobacteria, Bacteroides* and *Patiscibacteria*.

Among the HIV control cohort the dominant phyla were: *Firmicutes (36%), Actinobacteria (24%), Bacteroidetes (23%), Proteobacteria (16%)* and *Fusobacteria (4%).* In patients with pneumonia who had negative TB cultures, there was a dominance of the phyla: *Proteobacteria (28%), Firmicutes (18%), Actinobacteria (10%)* and *Bacteroides (10%)*.

The microbiome of patients with PTB was dominated by the following phyla: *Proteobacteria (32%)*, *Firmicutes(17%)*, *Actinobacteria(10%)* and *Bacteroidetes(10%)*. *Proteobacteria* and *Firmicutes* constituting nearly half of the population. Among the phylum *Proteobacteria* the most common families were: *Burkholderiaceae*, *Enterobacteriaceae* and *Neisseriaceae*. The families *Lachnospiraceae, Veilonellaceae*, *Peptostreptococcaceae*, and *Staphylococcaceae* were the most commonly occurring *Firmicutes*. Among the phylum *Actinobacteria* the most common families were: *Micrococcaceae, Microbacteriaceae,, Bifidobacteriaceae, Norcardia* and *Actinomycetaceae*. In the phylum *Bacteroidetes* the prominent families were *Prevotellaceae*.

**Figure 2:**
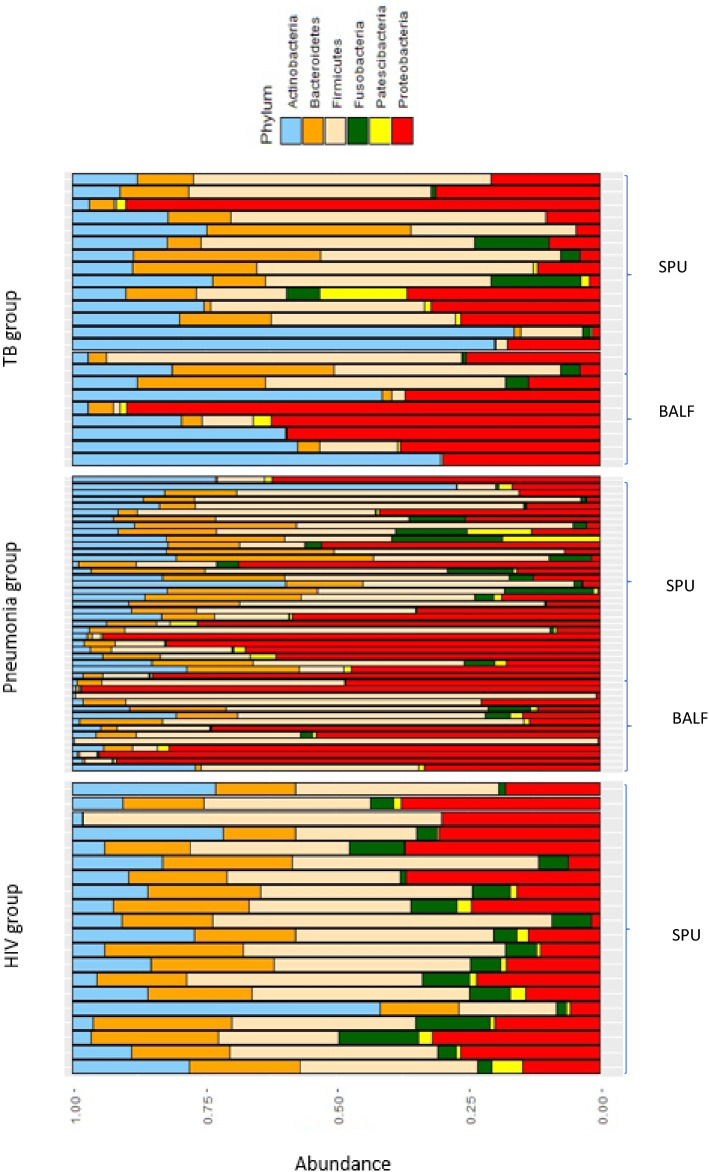
Relative abundances of the six dominant phyla between study cohorts, i.e., HIV, Pneumonia and TB across all specimens (sputum (SPU) and BALF).

**Figure 3:**
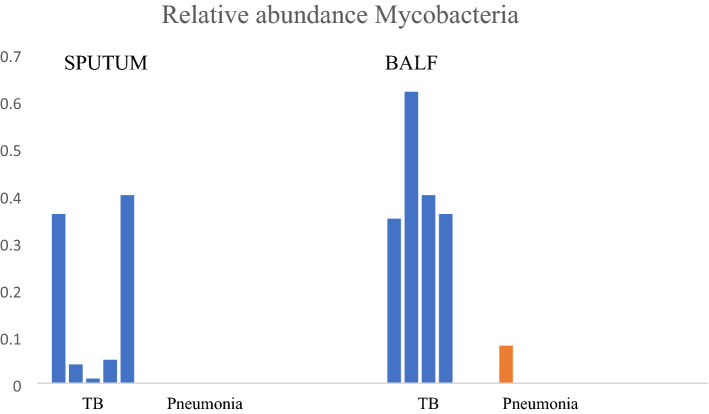
Relative abundance of mycobacteria in the various cohorts: HIV, Pneumonia and TB in sputum and in BALF.

Differences in the abundance of dominant phyla were detected between the study cohorts: the HIV cohort wase dominated by *Firmicutes* (36%) while communities in patients with pneumonia and TB were dominated by *Proteobacteria* (28% and 32% respectively). The relative abundances of the dominant phyla in the three cohorts studied is shown in Fig. 2.The relative abundance of *Mycobacterium* between cohorts is illustrated in Fig. 3. The fact that mycobacteria was over-represented in the cohort with pneumonia who were culture-negative for TB, was further investigated and found to be driven by a patient who had *Mycobacterium avium* on culture.

### Impact of different disease states on microbial diversity

**Figure 4:**
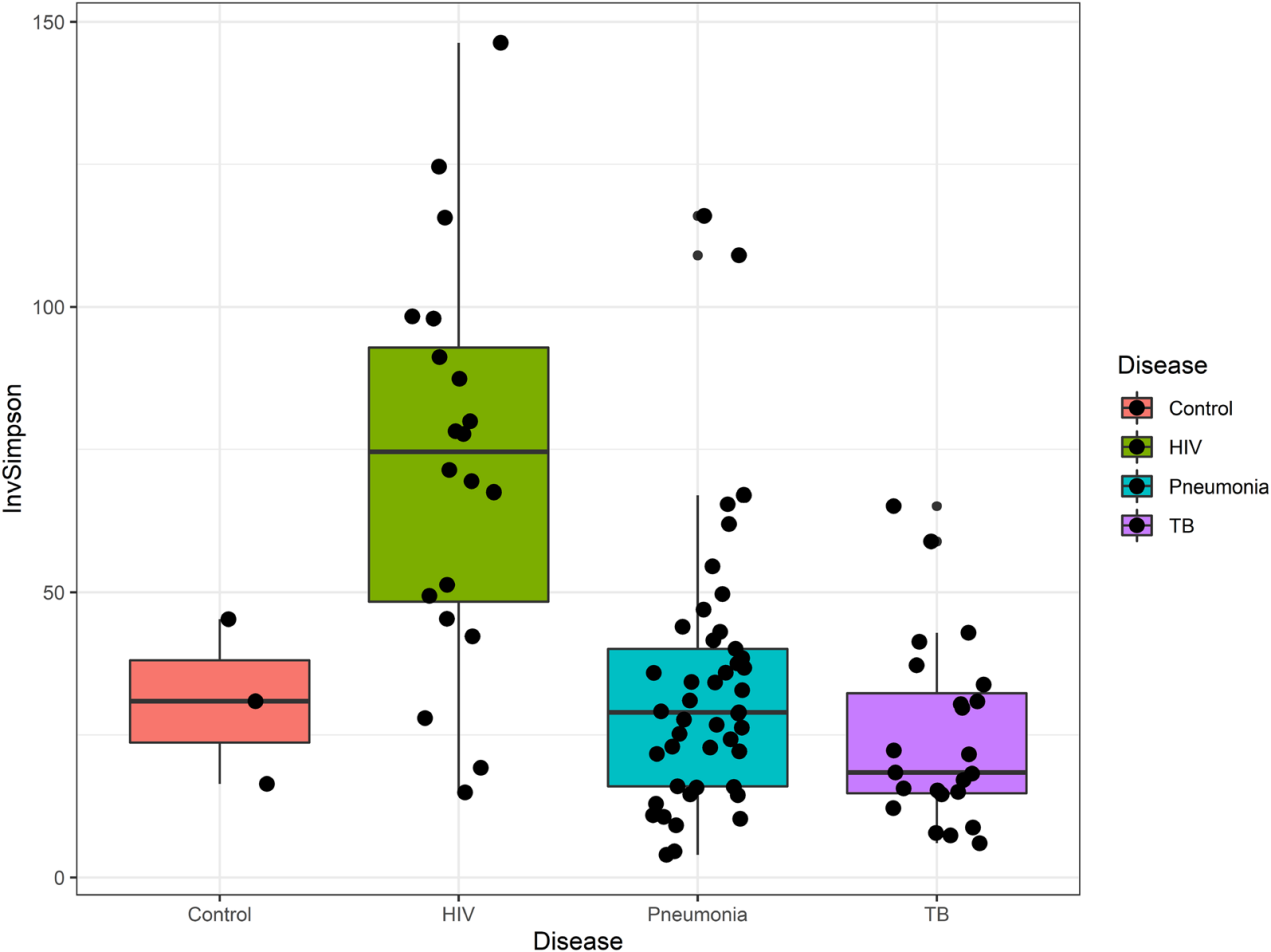
Box plot of the Inverse Simpson alpha diversity between cohorts the HIV (green), Pneumonia (blue) and TB (purple). The negative controls (red) were 0.9% saline samples.

A loss in microbial biodiversity (as represented by alpha-diversity indexes used in this study) was detected when in specimens (combined sputum and BALF) of patients with Pneumonia and those with TB when compared to the HIV cohort (Table 2, Fig. 4).

**Table 2:** Alpha diversity by the indices: Observed, Shannon and Inverse Simpson the between various cohorts.

**HIV cohort compared to the Pneumonia cohort**			
	**HIV (n = 20)**	**Pneumonia (n = 45)**	p value*
**Observed**			
Median (IQR)	279.5 (213.0–342.5)	130.0 (74.0–240.0)	< 0.001**
Min / Max	40.0/626.0	23.0/400.0	
**Shannon**			
Median (IQR)	4.9 (4.4–5.0)	3.8 (3.4–4.3)	< 0.001**
Min/max	3.2/5.4	1.6/5.2	
**Inverse Simpson**			
Median (IQR)	74.5 (47.4–94.6)	18.4 (14.6–33.8)	< 0.001**
Min/max	14.9/146.3	4.0 / 116.0	

**HIV cohort compared to the TB cohort**			
	**HIV (n = 20)**	**TB (n = 28)**	p value*
**Observed**			
Median (IQR)	279.5 (213.0–342.5)	100.0 (65.0–147.0)	< 0.001**
Min/max	40.0/626.0	24.0/299.0	
**Shannon**			
Median (IQR)	4.9 (4.4–5.0)	3.4 (3.0–3.9)	< 0.001**
Min/max	3.2/5.4	2.3/4.9	
**Inverse Simpson**			
Median (IQR)	74.5 (47.4–94.6)	18.4 (14.6–33.8)	< 0.001**
Min/max	14.9/146.3	6.0 / 65.1	

**Pneumonia cohort compared to the TB cohort**			
	**Pneumonia (n = 45)**	**TB (n = 28)**	p value*
**Observed**			
Median (IQR)	130.0 (74.0–240.0)	100.0 (65.0–147.0)	0.054
Min/max	23.0/400	24.0/299.0	
**Shannon**			
Median (IQR)	3.8 (3.4–4.3)	3.4 (3.0–3.9)	0.054
Min/max	1.6/5.2	2.3/4.9	
**Inverse Simpson**			
Median (IQR)	29.0 (16.0–40.1)–94.6)	18.4 (14.6–33.8)	0.101
Min/max	4.0/116.0	6.0/65.1	

*Nonparametric Wilcoxon rank sum test.

**Statistically significant.

Figure 4 illustrates the Inverse Simpson alpha diversity between the different cohorts.

**Figure 5:**
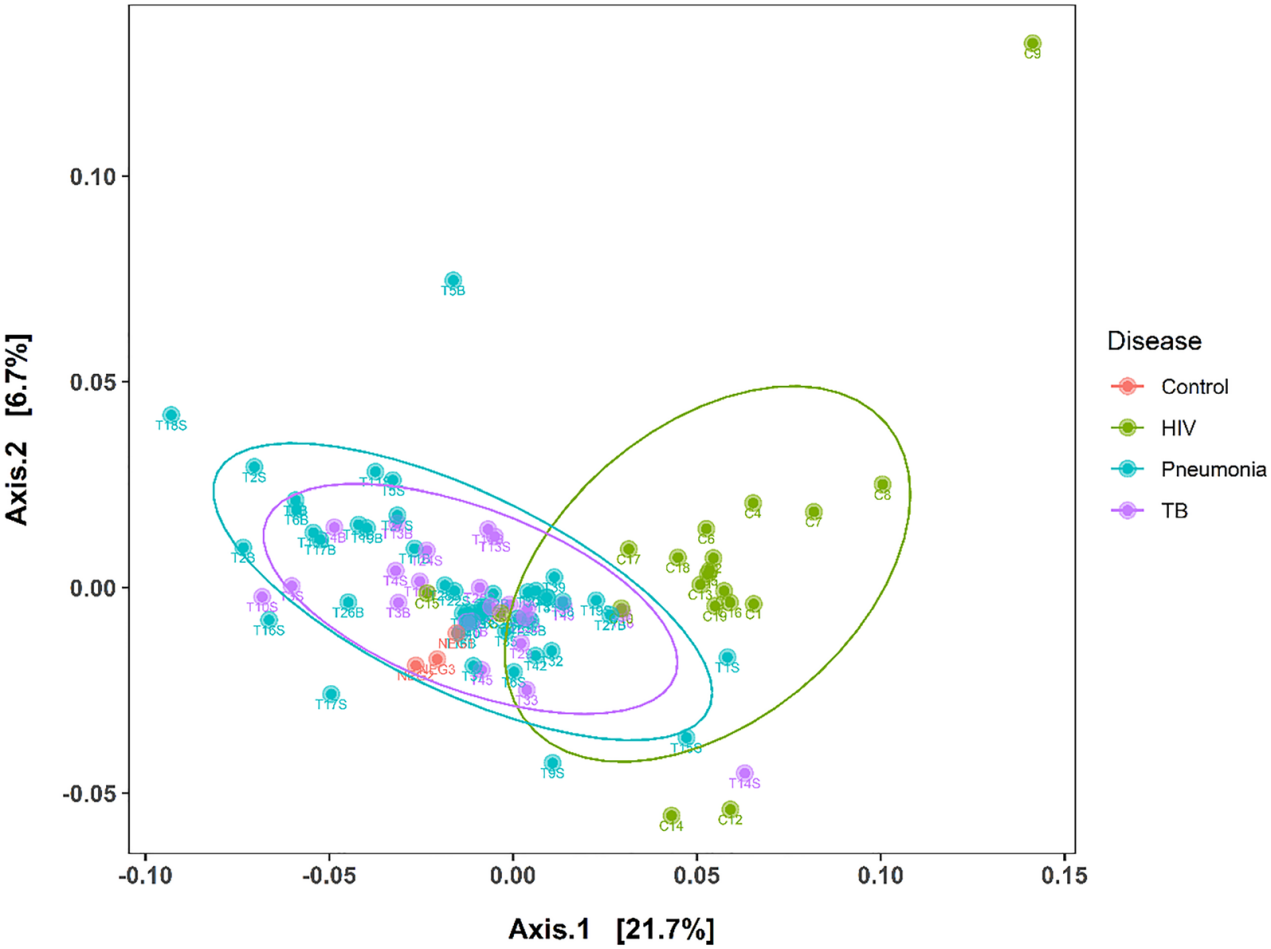
A PCoA Unirac unweighted analysis of inter- sample variability between the cohorts HIV (green), Pneumonia (blue), TB (purple) and negative controls (red) using the Bray–Curtis dissimilarity index. Principal coordinates 1 and 2 (labelled axis 1 and axis 2) explain 6.7% and 21.7% of the variance in Bray–Curtis dissimilarity, respectively.

To investigate differences in community structure between the cohorts the Bray–Curtis and Jaccard beta-diversity indexes of samples in each cohort were calculated and compared. The results from this analysis showed that the TB and pneumonia cohorts shared similar community structures, while being significantly distinct from the HIV cohort. A significant difference in beta diversity was found when comparing the TB cohort with the HIV cohort by abundance, according to the Bray–Curtis index (R^2^ = 0.118, p = 0.004) and in composition by the Jaccard index (R^2^ = 0.1, p = 0.005). These results are depicted in Fig. 5.

The analysis indicated that the lung microbial communities from TB and Pneumonia patients clustered together (i.e., microbial communities in in disease-state patients), while HIV microbial communities (cohort without respiratory disease) clustered separately, but with some degree of overlap.

### Differential abundance of clinically relevant taxa

To investigate further the identified differences in community structure between cohorts, differential abundance analysis was performed to identify taxa that were specifically enriched in each cohort. Over-representation of ≥ log twofold change was considered significant. The pneumonia cohort showed an over-representation of 6 genera when compared to the HIV cohort: *Achromobacter, Acinetobacter, Mycobacterium, Stenotrophomonas, Corynebacterium* and *Pseudomonas*.

Six of these taxa were also found to be over-represented in the TB cohort when compared with the HIV cohort, namely (in descending order of log change): *Achromobacter, Mycobacterium, Acinetobacter, Stenotrophomonas* and *Pseudomonas*. By comparison, in the HIV cohort the genera *Lautropia, Filifactor, Neisseria* and *Haemphilus* were over-represented when compared to the TB cohort. No differences in genera abundance could be identified between the TB and Pneumonia cohorts, which is consistent with the beta-diversity results showing a big overlap in community structure between these cohorts.

### Comparing BALF and sputum samples

Of the 71 patients enrolled in this study only 28 underwent bronchoscopy and BAL. To assess whether the different sampling methods had an impact on the microbial diversity and composition of the specimens the alpha diversity of the sampling strategies was compared.

**Figure 6:**
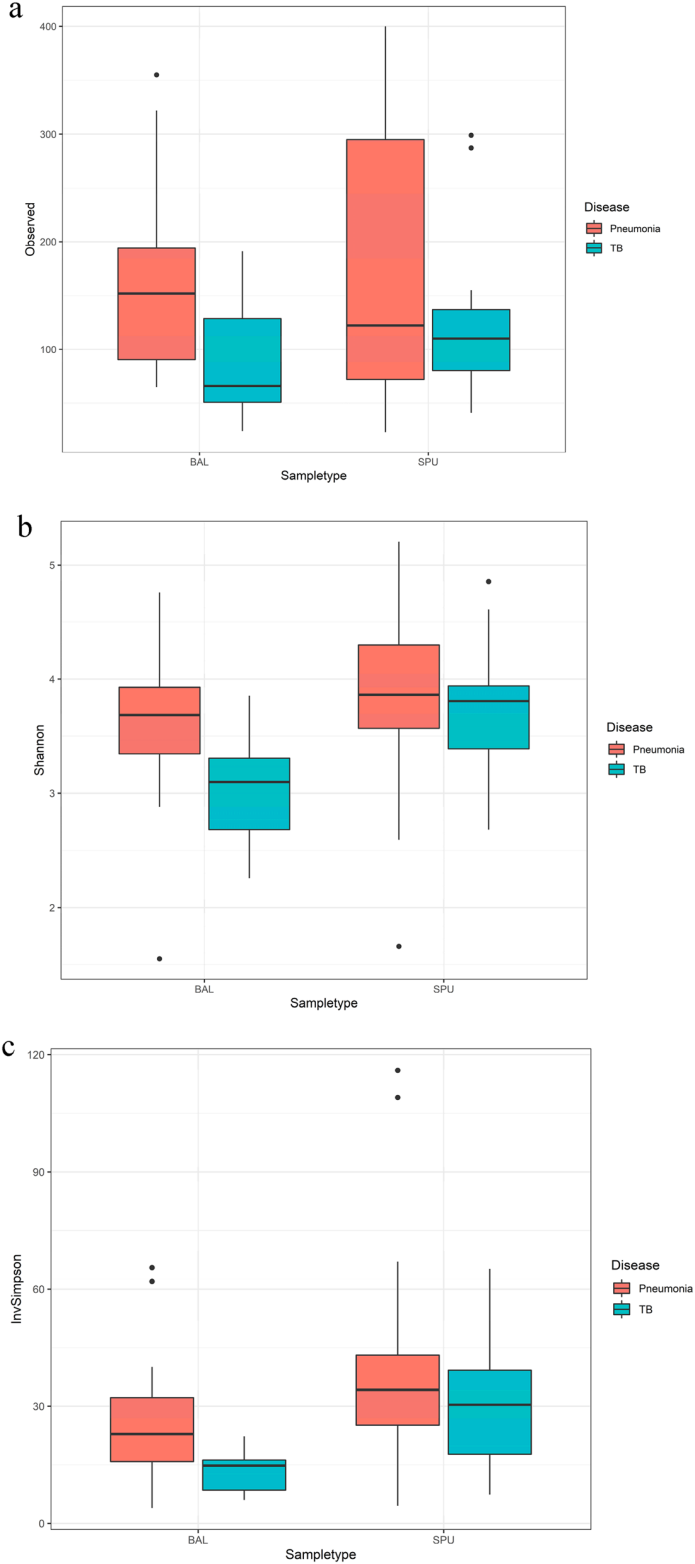
Alpha diversity in the BALF and sputum of Pneumonia and TB cohorts by Observed (**a**), Shannon (**b**) and Inverse simpson (**c**) indices.

When only BALF specimens were compared, there was a statistically significant difference with more pronounced dysbiosis in the cohort with TB, when the median values of the Shannon and Inverse Simpson diversity indices were considered (Observed: pneumonia 152, TB 55, p 0.067; Shannon: pneumonia 3.7, TB 3.1 p = 0.017; Inverse Simpson: pneumonia 22.9, TB 14.8. p = 0.014). These results are illustrated in Fig. 6.

Looking at the community structure, sampling strategy explained 10% of the dissimilarity between the community of the different specimens. Differential abundance analysis of the specimens obtained with the two sampling strategies identified seven genera that were differently abundant across specimens. In the BALF of the TB specimens, the genus *Stenotrophomonas* was over-represented when compared with the BALF specimens of the Pneumonia cohort. In the pneumonia cohort *Neisseria, Corynebacterium, Dialister* and *Prevotella* were over-represented.

## Discussion

This is the first report of the lung microbiome composition in a population of African patients with underlying HIV, with and without pneumonia. The lung microbiome of patients with TB in this study were dominated by *Proteobacteria, Firmicutes* and *Actinobacteria,* with a relatively smaller proportion of *Bacteroidetes* when compared to the HIV cohort. The decrease in frequency of *Bacteroidetes* when compared to *Proteobacteria* and *Firmicutes* has been observed in other pulmonary disease states, such as asthma^36^, where it is associated with severe inflammation.

As the majority of TB cases reported in South Africa occur in the context of HIV co-infection, the controls selected were HIV positive with no respiratory disease. The rationale for this was the fact that HIV in itself (particularly advanced disease as in the cohort studied) effects a change on the respiratory microbiome. In a previous study HIV infection was associated with increased abundance of *Streptococcus* whilst there was comparatively less *Flavabacterium, curvibacter, Rickettsia* and *Borellia* found^37^. Advanced HIV infection was associated with a loss of alpha diversity and greater beta diversity^37^.

In previous studies on the lung microbiome in TB patients, *Enterobacteriaceae*^17^*, Neisseriaceae*^22^ and *Sphingomodaceae*^18^ were also identified as dominant families. The dominance of the *Burkholderiaceae* family in the cohort with TB is a novel finding in this study. *Burkholderia* infection in the lung has been described mainly in the context of CF and chronic granulomatous lung disease, where it is associated with poor clinical outcomes^36,38^. Previous TB infection was reported in 25% (5/20) of those within the cohort with TB and there may have been underlying destructive lung disease (bronchiectasis) in some of the participants—*Burkholderia* has been described in non-cystic fibrosis bronchiectasis^38^. The degree of immunosuppression of the patients with TB included in this study (mean CD4 count 127 cells/mm^3^) could be a further pre-disposing factor to explain the abundance of the genus *Burkholderia*, but this needs to be further studied.

The *Enterobacteriaceae* detected in the specimens of patients with TB (*Klebsiella, Enterobacter, Serratia* and *Morganella*) are also commonly associated with nosocomial pneumonia^39^ and community-acquired pneumonia in patients with underlying lung pathology^40^. *Lachnospiraceae* was a prominent *Firmicute* in patients with TB. A recent publication associated the presence of this family with adverse outcomes in the critically ill^41^. *Veilonellaceae* and *Prevotellaceae* are anaerobic families that are found in the oral microbiota^42^ and when present in the lung microbiome of patients with airways disease, these bacteria are associated with an increased host inflammatory response^43^. *Veilonella* was lso a dominant family in a previous study of HIV-negative patients with TB^22^. Enrichment of the lung microbiome with *Veilonella* and *Prevotella* is associated with increased concentrations of arachidonic acid and interleukin-17, constituting a pro-inflammatory environment^43^. In HIV patients with pneumonia, increases in the relative abundance of *Prevotella* are associated with a unique metabolomic fingerprint (enriched with amino acid metabolites and monoacylglycerols), a pro-inflammatory milieu with increased IL-17A and an increased risk for mortality^44^.

*Mycobacteria* was not found on 16S rRNA sequencing of all the specimens that were culture positive for *M. tuberculosis*. The gold standard for diagnosis of *M. tuberculosis* is culturing, and 16S rRNA sequencing is not routinely used for diagnosis of PTB. Cheung and colleagues (Cheung et al., 2013) found that *M. tuberculosis* represented a very small relative abundance in patients where it was considered a causative microorganism^18^. The cell walls of mycobacteria, by virtue of the richness in fatty acids and waxes, are resistant to some DNA extraction processes^45^. As the aim of the study was to describe the microbiome associated with active pulmonary TB, rather than to diagnose *M. tuberculosis* itself, DNA extraction did not include a process of mycobacterial cell wall lysis and during sequencing no primers that are specific for the genus *Mycobacterium* were used.

Previous studies have observed a loss of alpha diversity in lung samples from patients with advanced HIV infection^46^, which is why the control cohort selected for this study was HIV-positive. The CD4 count of the HIV-positive control cohort was higher than that of the TB cohort, which if used as a surrogate for may indicate more advanced disease in the latter cohort (the stage of HIV has previously been found to be associated with a loss of alpha diversity in the lung microbiome)^11^. Active pulmonary tuberculosis in itself may cause a modest decrease in CD4 cell count^47^, so the difference may not be a pure reflection of the stage of HIV.

Beta diversity was different between the HIV cohort and the cohorts with respiratory disease, be it Pneumonia or TB. The Jaccard analysis considers the number of species present, while the Bray–Curtis considers abundance of species. There was over-representation of the *Lautropia, Filifactor, Neisseria* and *Hemophilus* genera in the HIV cohort. In the TB cohort, the dissimilarity was driven by the over-representation of the following genera: *Achromobacter, Mycobacterium, Acinetobacter, Stenotrophomonas* and *Pseudomonas*, all of which are typically considered pathogenic bacteria. *Lautropia* has previously been isolated in the lung microbiome of children with HIV^48^, where it was not associated with clinical disease. *Achromobacter* spp. (predominant genus with the TB cohort) are frequent pathogens in healthcare-associated infections and has been associated with CF^49^. The study was performed in a referral hospital and many patients have frequent exposure to healthcare facilities before presentation to an Academic centre, potentially exposing them to nosocomial pathogens.

The over-representation of mycobacteria in the Pneumonia cohort, when compared to the HIV cohort, is partially explained by the presence of *M. avium intracellularae* cultured in one patient. Three other patients were found to have *Mycobacterium* spp. but the species subtypes were not classified in this study. The mycobacteria identified by 16S rRNA sequencing in those who were culture negative for TB may, therefore, either represent mycobacteria other than *M. tuberculosis*, or alternatively represent patients with *M. tuberculosis* who were missed by conventional culture^50^. Culture-negative pulmonary TB can occur in the early stages of the disease^51^.

When the TB cohort was compared with the Pneumonia cohort, no bacteria were found to be over-represented in either cohort. Lung microbiota have been correlated with specific metabolic signatures in the BAL of HIV-positive patients^52^. Metabolomic analysis may be useful to delineate possible functional differences in the lung microbiome in TB when compared with pneumonia in future studies.

A statistically significant difference in alpha diversity was found when BALF samples were compared between the two disease states (eight BALF specimens in the TB cohort and 20 in the Pneumonia cohort), with a relatively lower alpha diversity in patients with *M. tuberculosis.* This held true for both the Shannon and Inverse Simpson indices. Although the dominant phyla (*Proteobacteria, Firmicutes* and *Actinobacteria*) were similar in both cohorts of patients, a loss of diversity was shown in those with TB when BALF was considered. This is a significant finding, as the primary target for TB is the lower airway^53^, which typically has low microbial biomass. BALF is superior to sputum for the detection of TB in patients with HIV and low CD4 counts^53^ and BALF are likely to better represent the microbiome in *M. tuberculosis*. The over-expression of S*tenotrophomonas* in the BALF of patients with TB, when compared with the BALF of those with pneumonia is an interesting finding. *Stenotrophomonas maltophillia* is a pathogen that is well-recognized in hospital-associated infections^54^, but it is also emerging as a community-acquired pathogen^55^ which is associated with significant morbidity and mortality in immunocompromised patients^56^. Cui and colleagues (2012) described *Stenotrophamonas* as a dominant constituent in the sputum microbiome of patients with PTB^57^. In the analysis of specimens from an explanted lung in COPD, *Stenotrophomonas* was found in the more distal part of the lung, whilst *P. aeruginosa* dominated the microbial composition more proximally^12^. The over-expression of *Stenotrophomonas* in the BAL of patients with active pulmonary TB and the potential networks between the two microbial species needs to be further investigated. A limitation of this study was the small sample size, which limits the generalizability.

This study considered the lung microbiome of patients with TB in the context of HIV-co-infection, which is particularly applicable to Sub-Saharan Africa. It was limited by the number of BALF specimens available for comparison with sputum, and the signal for differences in diversity may have been stronger if more BALF specimens were available. Further research is needed to prove the significance of Bcc in the lung microbiome of patients with HIV and TB. The metabolomic signatures of the lung microbiome, the importance of the virome and mycobiome, as well as functional relationships between microorganism in the lung microbiome warrant further research.

## Conclusion

The lung microbiome of patients with active PTB showed a dominance of the phyla: *Proteobacteria, Firmicutes* and *Actinobacteria*. When compared to the control population with HIV only, there was a decreased abundance observed in the phyla *Bacteroidetes;* a finding previously associated with pulmonary disease such as asthma and indicative of a pro-inflammatory milieu It is likely therefore, that in patients with TB a similar inflammatory state exists.

The abundance of *Burhkolderia* in this cohort of patients with PTB is a novel finding that should be further investigated and may be related to underlying destructive lung disease from previous infection with *M. tuberculosis*. In this study, there is clear evidence of dysbiosis in the lung microbiome of HIV-positive patients with active PTB. No distinct relationships of *M. tuberculosis* with other bacteria could be elucidated and metabolomic analysis may provide insight into functional relationships that may exist beyond mere species identification. Based on the results of this study the BALF is likely to be more representative of the lung microbiome in *M. tuberculosis* infection, as it retrieves bacteria from the site of disease. Furthermore, the dominance of *Stenotrophomonas* in the BAL of patients with PTB may be indicative of a functional relationship between these two bacteria and should be further studied by network analyses, growth rates and transcriptomics.

## Supplementary Information

Supplementary Information 1.

Supplementary Information 2.

Supplementary Information 3.

Supplementary Information 4.

## Data Availability

The datasets generated and analysed during the current study are available in the University of Pretoria data repository and can be accessed at https://doi.org/10.25403/UPresearchdata.19491317.v1.

## Abbreviations

BAL: Broncho-alveolar lavage
BALF: Broncho-alveolar lavage fluid
CD: Cluster of differentiation
CF: Cystic fibrosis
COPD: Chronic obstructive pulmonary disease
DNA: Deoxyribonucleic acid
HAART: Highly active antiretroviral therapy
HIV: Human immunodeficiency virus
*M. tuberculosis*: *Mycobacterium tuberculosis*
NGS: Next generation sequencing
PTB: Pulmonary tuberculosis
rRNA: Ribosomal ribonucleic acid
TB: Tuberculosis
