# Supplementary material for: The lung microbiome in HIV-positive patients with active pulmonary tuberculosis
Source: Sci Rep. 2022 May 28;12:8975. doi: 10.1038/s41598-022-12970-3 (PMC9148312; doi:10.1038/s41598-022-12970-3)
Supplement: Supplementary file 1 — Supplementary Information 1. [file 41598_2022_12970_MOESM1_ESM.docx]

**Inclusion and Exclusion Criteria**

Inclusion criteria for the study :

1. Signed informed consent
2. Males and females of the age ≥ 18 years
3. HIV positive – diagnosed previously or on the current admission with positive ELISA

serology or viral load

1. No anti-tuberculous treatment administered before admission
2. No antibiotics used in the 4 weeks prior to presentation. Allowance for the first dose

of antibiotics in hospital is made to align with the hospital’s policy of early appropriate

antibiotic therapy. Any patient with more than 6 hours between time of administration

of antibiotic and sample collection was excluded.

Exclusion criteria

1. Pregnancy
2. Unstable heart disease in the form of acute coronary syndrome or dysrhythmia
3. Significant hypoxia with a PaO2/FiO2 ratio of less than 200
4. Use of antibiotics within the 4 weeks prior to presentation
5. Known chronic lung disease in the form of cystic fibrosis, bronchiectasis, chronic obstructive airways disease or asthma.
6. Bleeding diathesis or laboratory evidence of possible hypocoagulability, with platelet count less than 150 000/µL or international normalized ration >1.5 or thromboelastographic evidence of hypocoagulability.
