# Supplementary material for: The lung microbiome in HIV-positive patients with active pulmonary tuberculosis
Source: Sci Rep. 2022 May 28;12:8975. doi: 10.1038/s41598-022-12970-3 (PMC9148312; doi:10.1038/s41598-022-12970-3)
Supplement: Supplementary file 2 — Supplementary Information 2. [file 41598_2022_12970_MOESM2_ESM.docx]

**Scripts in QIMME and R analysis**

**QIMME-2 SCRIPTS**

**1.1 Importing data**

qiime2@qiime2core2019-10:/media/sf_PhD_Data

--type EMPSingleEndSequnces \

--input-path emp-single-end-sequeneces \

--output-path emp-single-end-sequences.qza

**1.2 Demultiplexing Sequences**

qiime demux emp-single **\**

--i-seqs emp-single-end-sequences.qza **\**

--m-barcodes-file sample-metadata.tsv **\**

--m-barcodes-column barcode-sequence **\**

--o-per-sample-sequences demux.qza **\**

--o-error-correction-details demux-details.qza

A summary of the demultiplexed results was generated:

qiime demux summarize **\**

--i-data demux.qza **\**

--o-visualization demux.qzv

**1.3 Quality control – DADA2**

The DADA2 was the pipeline selected as the quality control method used to detect and correct (where possible) amplicon sequence data on the Illumina platform (Illumina, USA). A quality plot was generated and to obtain a quality score of >15 forward sequences were cut at position 8 and truncated at position 280, while reverse sequences were cut at position 8 and truncated at position 210.

The scripts were as follows:

qiime dada2 denoise-paired **\**

--i-demultiplexed-seqs demux.qza **\**

--p-trim-left-f 8 **\**

--p-trim-left-r 8 \

--p-trunc-len-f 280 **\**

--p-trunc-len-r 210 **\**

An OTU table was generated:

--o-representative-sequences rep-seqs-dada2.qza **\**

--o-table table-dada2.qza **\**

--o-denoising-stats stats-dada2.qza

Metadata was tabulated:

qiime metadata tabulate **\**

--m-input-file stats.qza **\**

--o-visualization stats.qzv

**1.4 Generation of a tree for phylogenetic diversity analyses**

To aid phylogenetic diversity metrics, a tree for phylogenetic diversity analysis was created with the following script:

qiime phylogeny align-to-tree-mafft-fasttree **\**

--i-sequences rep-seqs.qza **\**

--o-alignment aligned-rep-seqs.qza **\**

--o-masked-alignment masked-aligned-rep-seqs.qza **\**

--o-tree unrooted-tree.qza **\**

--o-rooted-tree rooted-tree.qza

**1.5 Taxanomic analysis**

Taxanomic analysis was done and related to sample metadata. The Silva classifier was

applied to sequences to generate taxonomy classification.

qiime feature-classifier classify-sklearn **\**

--i-classifier silva-132-99-515-806-nb-classifier.qza **\**

--i-reads rep-seq.qza **\**

--o-classification taxonomy.qza

qiime metadata tabulate **\**

--m-input-file taxonomy.qza **\**

--o-visualization taxonomy.qzv

**SCRIPTS IN R**

Analyses were performed in R version 3.6.3 (2020). The OTU tables were imported, and a taxonomy table created with the variables phylum, class, order, family, genus and species. Data was filtered to remove eukaryotic variables.

Relative abundance was calculated of the top six phyla represented in the three groups. This was calculated using percentages of clustered phyla. The subset was filtered to include only those phyla that appear in 90% of specimens and only where the taxa represented more than 1% of the total.

The steps and scripts for R-suite were as follows:

**1.1 To set working folder**

> setwd(“C:/Users/uecke/OneDrive/Desktop/PhD Data”)

1.2 To install packages

install.packages("vegan", repos="http://cran.rstudio.com/", dependencies=TRUE)

install.packages("ggplot2", repos="http://cran.rstudio.com/", dependencies=TRUE)

install.packages("RColorBrewer", repos="http://cran.rstudio.com/", dependencies=TRUE)

if (!requireNamespace("BiocManager", quietly = TRUE))

install.packages("BiocManager")

BiocManager::install("phyloseq")

**1.3 To activate packages**

library(vegan)

library(ggplot2)

library(RColorBrewer)

library(RAM)

library(gridExtra)

library(cowplot)

library(phyloseq)

**1.4 Import OTU & metadata tables**

#importing the bacterial data as data.frame objects

otu_table = read.table (file.choose(), header = TRUE, row.names = 1 , sep = ";")

#because of formatting, the normal read.table function does not work for taxonomy. instead, use the import dataset menu (choose import text)

#after that, convert first column into row names

rownames(taxonomy) = taxonomy[,1]

#delete first column, which will have duplicated names

taxonomy[,1] = NULL

metadata = read.table (file.choose(), header = TRUE, row.names = 1, sep = ";")

#converting taxonomy and otu table into matrix class objects

otu_table_matrix = as.matrix(otu_table)

class(otu_table_matrix)

taxonomy_matrix = as.matrix (taxonomy)

#converting all data objects into phyloseq objects

otu_Lung = otu_table (otu_table_matrix, taxa_are_rows = TRUE)

tax_lung = tax_table(taxonomy_matrix)

sampledata_lung = sample_data(metadata)

physeq_Lung = phyloseq(otu_Lung, sampledata_lung, tax_lung)

physeq_Lung

**1.5 Filtering taxa**

physeq_Lung = subset_taxa(physeq_Lung, tax_table(physeq_Lung) != "Eukaryota")

#filtering samples

physeq_Lung_2 = subset_samples ( physeq_Lung, sample_names(physeq_Lung) != c("JG.1515756"))

#Cluster all your OTUs into phylum level

physeq_Lung_RA_2 = transform_sample_counts(physeq_Lung_2, function (x) x/sum(x))

physeq_Lung_phylum = tax_glom (physeq_Lung_2, taxrank = "Phylum")

Mycobacteria = subset_taxa(physeq_Lung_RA_2, Genus == "Mycobacteria")

physeq_dominant= prune_samples(sample_sums(physeq_Lung_2)<= 10000, physeq_Lung_2)

#Relative abundance graph bact

#transform counts of table to relative abundance

physeq_Lung_RA = transform_sample_counts(physeq_Lung_phylum, function (x) x/sum(x))

In this study filtering taxa that represent more than 1 percent of total sample count were filtered.

physeq_dominant= filter_taxa(physeq_Lung_RA, function(x) mean(x) > 0.01 , TRUE)

RA_bact = plot_bar(Stenotrophomonas, fill = "Genus") + labs(x="Sample", y = "Abundance") + facet_wrap(~Disease, scales = "free_x", nrow = 1)

RA_bact_3 = RA_bact + scale_fill_manual(values = c("Actinobacteria" = "lightskyblue", "Bacteroidetes" = "orange", "Firmicutes" = "moccasin", "Fusobacteria" = "darkgreen", "Patescibacteria" = "yellow1", "Proteobacteria" = "red"))

ggsave(filename = "Steno_relative_abundance.png", RA_bact, width = 20, height = 12, dpi = 900)

#calculate alpha_diversity measures

alpha_diversity = estimate_richness(physeq_Lung_2)

#extract alpha_diversity table

write.csv(alpha_diversity, "alpha_diversity.csv" )

#import new alpha diversity table with a column with cohort groupings

alpha_div_2 = read.table (file.choose(), header = TRUE, row.names = 1 , sep = ";")

#subset your data.frame

TB_Pneumonia = subset(alpha_div_2, alpha_div_2$Disease == "Pneumonia"| alpha_div_2$Disease =="TB")

#subset your data.frame

SPU = subset(TB_Pneumonia, TB_Pneumonia$Sampletype == "SPU")

#check for normalized alpha_diversity to know what test to run

shapiro.test(TB_Pneumonia$Shannon)

hist(alpha_diversity$Observed, main="InvSimpson", xlab="", breaks=15)

# calculate significant difference between the cohorts

#For normally distributed data

anova_shannon = aov(Shannon ~ Disease, data = SPU)

summary(anova_shannon)

#For not normally distributed data

Wallis_Observed = kruskal.test(Observed ~ Disease, data = SPU)

Wallis_InvSimpson = kruskal.test(InvSimpson ~ Disease, data = SPU)

#calculate differences between more than two groups

#for normal data

Tukey_normal = TukeyHSD(anova_shannon)

#for non-normal data

InvSimpson_Wilcox = pairwise.wilcox.test(alpha_div_2$InvSimpson, alpha_div_2$Disease, p.adjust.method="fdr")

Observed_Wilcox = pairwise.wilcox.test(alpha_div_2$Observed, alpha_div_2$Disease, p.adjust.method="fdr")

#plot the alpha diversity between groups

p <- ggplot(TB_Pneumonia, aes(x=Sampletype, y= InvSimpson,fill= Disease)) +

geom_boxplot()

p2 = p3 = p + theme_bw()

ggsave(filename = "alpha_InvSimpson_BAL_SPU.png", p3, width = 8, height = 6, dpi = 900)

**1.6 Beta diversity calculations**

​1.6.1 **Filtering**​

​ physeq_Lung_2 = subset_amples ( physeq_Lung, sample_names(physeq_Lung) != c("JG.1515756"))

physeq_pruned_bac_10 = filter_taxa(physeq_pruned_bac, function(x) sum(x) > 10, TRUE)​

physeq_pruned_fungal_10 = filter_taxa(physeq_lung_2, function(x) sum(x) > 10, TRUE)​

sample_data(physeq_transformed_b) = sample_data(metadata_b)​

physeq_b_trimmed = subset_samples (physeq_transformed_b, sample_names(physeq_ACE_f) != "SG.17.111")​

​erie_PCoA <- ordinate(​

  physeq = physeq_transformed_b , ​

  method = "PCoA", ​

  distance = "bray"​)​

summary (erie_PCoA)​

​

​p1 = plot_ordination(​

  physeq = physeq_transformed_b,​

  ordination = erie_PCoA,​

  color = "Site", axes = 1:2) + theme_bw() +scale_color_manual(values= manual)​

p2 = p1 +  geom_point(size = 6, alpha = 1/2) + theme(axis.text.x = element_text(face="bold", size=10),axis.text.y = element_text(face="bold", size=10))​

p3 = p2 + theme(axis.title.x = element_text(size=12, face="bold", margin = margin(t = 20,r = 20, b = 20,l = 0)),axis.title.y = element_text(size=12, face="bold", margin = margin(t = 0, r = 20, b = 0, l = 20)))​

p3 = p3 +  theme(panel.grid.major = element_blank(), panel.grid.minor = element_blank())​

p3​

summary(p3)​

ggsave(filename = "PCOA_bacteria_rarefy_bray_MA.png", p3, width = 8, height = 6, dpi = 900)​

​

erie_PCoA_un= phyloseq::distance(physeq_transformed_b, method = "bray")​

summary(erie_PCoA_un)​

sampledf <- data.frame(sample_data(physeq_transformed_b))​

adonis(erie_PCoA_un ~Site, data = sampledf, permutations = 1000)​

summary(adonis)​

beta <- betadisper(erie_PCoA_un, sampledf$Site)​

permutest(beta)​

​plot(beta)​

TukeyHSD(beta)​

​

physeq_b_rarefy = rarefy_even_depth(physeq_pruned_fungal_10, rngseed = 1, sample.size = 1*min(sample_sums(physeq_pruned_fungal_10)), replace = F)​

​

physeq_transformed_f = transform_sample_counts(physeq_b_rarefy, function (x) log(x+1))​

​

erie_PCoA <- ordinate(​  physeq = physeq_transformed_f, ​method = "PCoA", ​distance = "bray"​)​

​p1 = plot_ordination(​

  physeq = physeq_transformed_f,​

  ordination = erie_PCoA,​

  color = "Site", axes = 1:2) + theme_bw() +scale_color_manual(values= manual)​

p2 = p1 +  geom_point(size = 6, al

pha = 1/2) + theme(axis.text.x = element_text(face="bold", size=10),axis.text.y = element_text(face="bold", size=10))​

p3 = p2 + theme(axis.title.x = element_text(size=12, face="bold", margin = margin(t = 20,r = 20, b = 20,l = 0)),axis.title.y = element_text(size=12, face="bold", margin = margin(t = 0, r = 20, b = 0, l = 20)))​

p3 = p3 +  theme(panel.grid.major = element_blank(), panel.grid.minor = element_blank())​

p3​

​

ggsave(filename = "PCOA_fungal_rarefy_bray.png", p3, width = 8, height = 6, dpi = 900)​

​

erie_PCoA_un= phyloseq::distance(physeq_transformed_b, method = "bray")​

sampledf <- data.frame(sample_data(physeq_transformed_f))​

adonis(erie_PCoA_un ~ Site, data = sampledf, permutations = 1000)​

​

beta <- betadisper(erie_PCoA_un, sampledf$Site)​

permutest(beta)​

​

TukeyHSD(beta)​

​

write.csv(table_b, "table_bacteria_transposed.csv")​

​

table_b = as.data.frame(t(otu_table(physeq_pruned_bac_10)))​

write.csv(table_b, "table_bacteria_transposed.csv")​

write.csv(tax_table(physeq_pruned_bac_10), "tax_bacteria_transposed.csv")​

​table_b_transposed=read.table (file.choose(), header = TRUE, row.names = 1 , sep = ",")​

metadata=read.table (file.choose(), header = TRUE, row.names = 1 , sep = ",")​

​​(sim <- with(metadata, simper(table_b_transposed, Site)))​

table_B_simper=summary(sim)​

​simper = as.data.frame(table_B_simper)​
