# Supplementary material for: The lung microbiome in HIV-positive patients with active pulmonary tuberculosis
Source: Sci Rep. 2022 May 28;12:8975. doi: 10.1038/s41598-022-12970-3 (PMC9148312; doi:10.1038/s41598-022-12970-3)
Supplement: Supplementary file 4 — Supplementary Information 4. [file 41598_2022_12970_MOESM4_ESM.docx]

**Over-representation of microorganisms at genus level in the BALF of patients with TB**

**compared to those with Pneumonia**
